# Critical analysis of the scientific production on Jean Watson’s Theory of Human Care

**DOI:** 10.1590/0034-7167-2023-0231

**Published:** 2024-06-14

**Authors:** Shirley da Rocha Afonso, Maria Itayra Padilha, Vanessa Ribeiro Neves, Noe Ramírez Elizondo, Ricardo Quintão Vieira

**Affiliations:** I Universidade Federal de São Paulo. São Paulo, São Paulo, Brazil; II Universidade Federal de Santa Catarina. Florianópolis, Santa Catarina, Brazil; III Universidad de Costa Rica. San José, Costa Rica

**Keywords:** Nursing Care, Nursing Theory, Nursing Education, Models of Nursing, Methods, Atención de Enfermería, Teoría de Enfermería, Educación en Enfermería, Modelos de Enfermería, Métodos

## Abstract

**Objectives:**

to analyze current scientific production on the use of the theoretical-methodological precepts of Jean Watson’s Theory of Human Care/Clinical Caritas Process.

**Methods:**

quantitative, exploratory, descriptive study using data from 1979 to 2023 in the Scopus database. Analysis was carried out using VOSviewer software.

**Results:**

the 73 studies included establish a network of collaboration among 221 authors from 155 institutions in 18 countries, who discuss the development of ethical behavior in nursing staff, through technical improvement, implementation, and validation of instrumental strategies capable of measuring and evaluating the quality of holistic and empathetic care. The Theory of Human Caring contributes to nursing training and care, and Process Clinical Caritas-Veritas is useful for the different possibilities of practice and education.

**Conclusions:**

it is important to strengthen with more empirical data a nursing work model centered on individual human care, supporting the evolution of scientific nursing knowledge.

## RESUMEN

**Objetivos:**

analizar la producción científica actual sobre la utilización de los preceptos teórico-metodológicos de la Teoría del Cuidado Humano/Proceso de Cáritas Clínica de Jean Watson.

**Métodos:**

estudio cuantitativo, exploratorio y descriptivo usando datos de 1979 hasta 2023 en la base de datos *Scopus*. Análisis mediante el programa *VOSviewer*.

**Resultados:**

los 73 estudios incluidos establecen una red de colaboración entre 221 autores de 155 instituciones de 18 países, que discuten el desarrollo del comportamiento ético en el personal de enfermería, a través de la mejora técnica, implementación y validación de estrategias de instrumentos capaces de medir y evaluar la calidad del cuidado holístico y empático. La Teoría del Cuidado Humano contribuye a la formación y cuidados de enfermería y el Proceso Clínico Caritas-Veritas es útil para las diferentes posibilidades de práctica y educación.

**Conclusiones:**

es importante fortalecer con más datos empíricos un modelo de trabajo enfermero centrado en el cuidado humano individual, subsidiando la evolución del conocimiento científico enfermero.

## INTRODUCTION

Knowledge production is linked to the need to develop references capable of guiding the path of thought. This contemplates a distinct action capable of consolidating relationships between those who produce knowledge and those who will benefit through care actions. Nursing is the profession of care, and this is the basis of action developed in all healthcare environments around the world^(1)^.

The theoretical-philosophical bases, values and worldviews transcend specific issues and seek to provide universal understanding of reality, in addition to ethical-philosophical reflection for all fields of study^(2)^. Thus, it is understood that nursing configures its knowledge with the multiplicity of knowledge production.

Nursing theories point to the construction of this multiple knowledge, providing theoretical and practical material for carrying out daily care actions. Validated knowledge contributes to the advancement of the profession, as it fills the gap in debates and reflections on nursing practice. Theories and concepts consolidate the conceptual interrelations of knowledge itself, establishing and determining a logical relationship of critical thinking^(3, 4)^.

Over the last 68 years, several nursing scholars have established concepts and theories to explain, provide the necessary tools and systematize the understanding of care in daily nursing practice, such as Virginia Henderson (Need Theory, 1955), Faye Abdellah (21 Nursing Problems Theory, 1960), Ernestine Wiedenbach (Prescriptive Theory, 1964), Imogene M. King (Theory of Goal Attainment, 1964), Sister Callista Roy (Adaptation Model of Nursing, 1964), Lydia Hall (Care, Cure, Core Nursing Theory, 1966), Josephine Paterson and Loretta Zderad (Humanistic Nursing Theory, 1966), Myra Estrin Levine (Conservation Model of Nursing, 1967), Wanda de Aguiar Horta (Theory of Basic Human Needs, 1970), Martha Rogers (Science of Unitary Human Beings, 1971), Dorothea Orem (Self-Care Deficit Theory, 1971), Betty Neuman (Neuman System Model, 1971), Madeleine Leininger (Transcultural Nursing Theory, 1978), Jean Watson (Theory of Human Caring, 1979), among others^(5, 6, 7, 8, 9, 10, 11)^.

Among these, the focus of this study is the thought and assumptions defended by Jean Watson. Recognizing the actions disciplined by theories as an intrinsic activity of the nursing profession is admitting the establishment of interpersonal relationships in work routine and, with this, building social, emotional and spiritual experiences between nurses and patients.

Watson’s philosophy is based on a belief that caring is a moral ideal rather than a task-oriented one and that people take care of themselves to care for others. Watson sees nursing as a profession and caring as a vocation based on an ethical agreement linked to humanity^(12)^. She also considers care as something innovative, capable of strengthening nurses’ practice and leadership in the health field. Furthermore, her care proposal based on body-mindsoul integration is necessary as a tool to support patients’ and families’ experience and understanding about the disease and healing process^(13)^. Care advocated by Watson requires a deep connection between nurses and patients throughout every moment of care.

Care is transpersonal and provides the opportunity to explore feelings and emotions beyond technical-procedural nursing actions.

Relationships are interpersonal and promote the bond between body and mind based on “caring-healing” perception^(14)^. In other words, experiences acquired through interpersonal relationships between nurses and patients transcend the perception of being, and acquire consciousness connected with persons cared for and persons who care. It is an intersubjective idea that shares the both participants’ life story in the moment of care^(13, 14, 15, 16)^. The Theory of Human Caring is based on a system of values built on a holistic perspective and a unitary worldview that refers to a transpersonal experience of the body, spirit and soul, created at the moment of care, and interpersonal relationships between nurses and patients. From this perspective, Jean Watson guides transpersonal care through the recognition of the “healing” awareness. This awareness, called Caritas, is a unity that means “being present in the moment of care”^(13, 14, 15, 16)^.

This understanding of genuine care in a true moment becomes evident in the evolution of the theory from 1980 onwards. In 1985, the first paradigm consisted of care based on factors formed by a humanistic-altruistic value system^(16)^. The second paradigm, in 2008, shows an even greater approach to care when it begins to assume authentic awareness through elements that support the transpersonal relationship and, then, expands to Clinical Caritas Process^(12)^. After ten years, Jean Watson presents her third paradigm, which involves nurses’ transcendence to an evolved awareness, open to the cosmic-divine and to love, adding key elements in more authentic care and expanding to Caritas-Veritas Clinical Process. In this third paradigm, Jean Watson states that care is a unique phenomenon and recognizes that it must occur in a unitary manner and, thus, proposes an expansion of understanding about the evolution of the Theory of Human Caring to Science of Unitary Care. In this regard, the perspective of care establishes a way of perceiving the world and relating in a way that emphasizes the transformative meaning of caring and being cared for, i.e., recognizing the interaction of the relationship as a unitary experience of thought, body, soul and spirit during the moment of care^(17)^.

Bearing this in mind, the guiding question arose: what is the overview of international scientific production regarding Jean Watson’s Theory of Human Caring? It is important to highlight that, by understanding this overview, it will be possible to preserve and convey technical-scientific knowledge about holistic and empathetic care.

The justification for this study is the understanding that the purpose of gathering experiences about research on human care will help in proposing new perspectives, given the evolution of the worldview and relationships between people that the theory presents. Furthermore, it will guide new research through the production of important scientific knowledge for the evolution of nursing practice based on unitary and intentional theory, emphasizing the connection of care experiences between nurses and patients.

## OBJECTIVES

To analyze current scientific production regarding the use of the theoretical-methodological precepts of the Theory of Human Caring/Clinical Caritas Process created by Jean Watson.

## METHOD

### Ethical aspects

The study used secondary documentary sources indexed in a database and, therefore, did not require approval from the Research Ethics Committee.

### Study design

This is a study with quantitative, exploratory and descriptive characteristics, with a bibliometric analysis design^(18)^. In this study, a hybrid model was applied through the systematization of a set of phases and stages, seeking to investigate the overview, applications and results obtained. Thus, the study was divided into two phases: 1. Search in the database; and 2. Bibliometric analysis.

### Data collection and organization

Data collection was carried out in the Scopus database, using the PRISMA 2020 Statement to present the definition of selected scientific articles systematically^(19)^. The search was carried out in March 2023. The intention to search in a single database was because Scopus can be considered a broader, more democratic database, containing a greater number of qualified journals than in other databases.

Although it follows the guidance of a bibliometric study, it was decided to search for systematic review based on a research question. The PCC strategy was adopted using the acronym for Population (Nursing), Concept (Theoretical-methodological precepts) and Context (Jean Watson’s Theory of Human Caring), with the concepts and contexts of the following terms being identified in the abstracts of the studies to be selected: Carative Factors, Watson’s Model, Watson’s Caring Science, Watson’s Human Caring Science, Watson’s Unitary Caring Science, Watson’s Theory of Caring or Watson’s Theory of Human Caring (Jean Watson) or Theory of Transpersonal Caring (Watson or Jean Watson). When considering the historical context, we chose to search for terms that expressed the implementation of the theory from 1979 to 2023, taking into account the various conceptions adopted over time. Inclusion criteria included original scientific articles with an approach to patient care based on the Theory of Human Caring, the relationships between the theory and nursing training, and the processes of implementing the theory in nursing work.

Exclusion criteria included conditions in which the theory was used only to compare with another nursing theory, conceptual approach to the theory of care, but without a link to the theory defended by Jean Watson, repeated work, review research (secondary research) and work without presenting abstracts.

**Figure 1:**
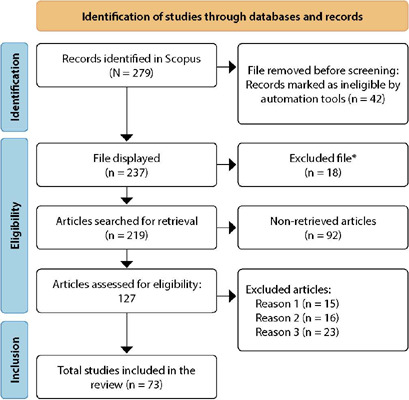
PRISMA 2020 flowchart for new systematic reviews that included searches only in databases and records

Source: PRISMA 2020.

The first stage of identification consisted of using the search strategy TITLE-ABS-KEY (Watson’s AND theory AND of AND caring) AND (LIMIT-TO (DOCTYPE, “ar”)). The term “Watson’s Theory of Caring” was restricted to the title, abstract or keywords fields to find 279 indexed documents in the Elsevier report indexed in Scopus until the date of information extraction, on March 25, 2023. After implementing the inclusion and exclusion criteria, 73 articles remained for the analysis of scientific production presented in Figure 1.

### Data analysis

The difference between the number of documents located and articles included in the analysis highlights PRISMA methodology’s relevance to produce a systematic review and analyzes using bibliometric indicators, as it allows minimizing the emergence of biases. Data analysis was initially carried out using the bibliometric method, which identified the most comprehensive setting in the literature, outlining the evolution and development of the topic studied. In this case, it was carried out using quantitative indicators from descriptive statistical sample to create graphic elements. Data were exported in “*.csv” format for bibliometric analysis in software, in order to treat them statistically by bibliometric networks in VOSviewer version 1.6.9^(18, 20)^. To organize qualitative information, data were tabulated in a Microsoft Excel 365 file, identifying abstracts, authorship, year of publication, institutional affiliation, journalo, method used and theoretical approach. This organization constituted an overview of systematic analysis, allowing the identification of gaps and trends in scientific production.

## RESULTS

Initially, works published from 1979 to 2023 were identified, however, after reading the abstracts, those that addressed the Theory of Human Caring/Clinical Caritas Process as the scope of the investigation were selected, which restricted selection to 2000 to 2022. There was no indexing of works in Scopus in 2005 and 2007; however, it is not possible to state the reason for the lack of research on the topic in journals during this period. An increase in the number of studies in indexed journals was identified in 2020 and 2021, highlighting a trend in the discussion about the theory. The year 2020 concentrated 11 studies in different journals^(21, 22, 23, 24, 25, 26, 27, 28, 29, 30, 31)^. And 2021 recorded one study in each journal, with *Revista Brasileira de Enfermagem* standing out with three articles^(32, 33, 34, 35, 36, 37, 38)^.

**Figure 2:**
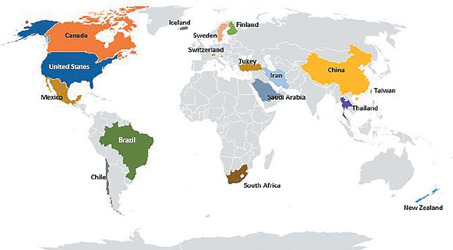
Geographic distribution of authors who developed studies related to Watson’s Theory of Human Caring

Of the 73 articles available with full access, it was possible to observe 221 associated authors, 155 institutions in 18 countries, as shown in Figure 2.

Figure 2 shows the countries of origin of the authors who published on the topic, such as the United States, with 56 authors, highlighting the University of Colorado, with eight authors; Brazil, with 51 authors, with emphasis on the *Universidade Federal da Bahia*, with nine authors; Turkey, with 25 authors; Canada, with 17 authors; China, with 15 authors; Chile and Switzerland, with 12 authors each country; South Africa, with six authors; Mexico, with five authors; Saudi Arabia, with four authors; Slovenia, Iran and Taiwan, with three authors each country; Iceland, Thailand and Finland, with two authors in each country; and New Zealand, with one author. It was possible to identify the temporal evolution of studies on the researched term, highlighting the number of occurrences recorded in each year from 2000 onwards, the first being published by Caroline L. Norred^(38)^.

Of the 221 authors, around 88% (195 authors) published only one article, while 11% (25 authors) published two to three articles. The United States of America is a country with the largest volume of authors with studies on the subject of study, totaling 26 articles indexed in Scopus, with emphasis on Jean Watson (eight articles)^(21, 39, 40, 41, 42, 43, 44, 45, 46)^.

The 73 articles that made up the sample are distributed across 47 journals. When observing dispersion of scientific production on Watson’s Theory of Human Caring, it was decided to arrange the journals into three groups, considering the total number of studies in each. The first group is made up of journals with a range of three and eight studies, and the second group has two studies each. The third group is made up of journals that include one study each. Chart 1 highlights the publication sources with the highest volume of articles in the investigated sample.

**Chart 1:** Distribution of the main journals that published works on Watson’s Theory of Human Caring, São Paulo, São Paulo, Brazil, 2023

Journals		Number of articles	Location
**Group 1 – 5 journals**		38	
Nursing Science Quarterly^(28, 29, 41, 42, 46, 47, 48, 49, 50, 52, 53, 54, 55, 56, 57, 58, 59)^		8	United States
Journal of Advanced Nursing^(35, 40, 50, 51, 52)^		5	United States
Journal of Holistic Nursing^(42, 53, 54, 55, 56)^		5	United States
*Ciencia y Enfermería* ^(47, 48, 49, 50, 51, 52, 53, 54, 55, 56, 57, 58, 59)^		3	Chile
*Revista Brasileira de Enfermagem* ^(37, 38, 60)^		3	Brazil
**Group 2 – 7 journals**		14	
AORN Journal^(39, 61)^		2	United States
Aquichan^(62, 63)^		2	Chile
BMC Nursing^(33, 64)^		2	Switzerland
Journal of Nursing Research^(65, 66)^		2	Saudi Arabia
*Nursing Fórum* ^(67, 68)^		2	United States
*Revista da Escola de Enfermagem da USP* ^(69, 70)^		2	Brazil
Scandinavian Journal of Caring Sciences^(23, 71)^		2	United States
**Group 3 – 35 journals**		35	
**Total**	47 journals	73 articles	

In relation to the journals with the highest publication volume, Nursing Science Quarterly stands out, which accounts for 33.3% of group 1 of studies in the study sample, followed by Journal of Advanced Nursing and Journal of Holistic Nursing, with 20.83% each, and *Ciencia y Enfermería* and *Revista Brasileira de Enfermagem*, which account for 12.5% each of the journals with the largest volume of published works. The other journals in group three total 14.28% each. Groups 1 and 2 represent 52.05% of the publication volume of the analyzed sample^(72, 73, 74, 75, 76, 77, 78, 79, 80, 81)^.

Chart 2 lists the most influential publications on the topic, considering the volume of citations received by other articles published in journals indexed in Scopus. The minimum number of 35 citations made among articles’ internal citation network was used as a cut-off criterion, resulting in eight articles that met this criterion, such as:

**Chart 2:** Volume of citations in articles with the greatest impact, São Paulo, São Paulo, Brazil, 2023

Author(s) and year	Title	Journal	Number of citations	Location
Mason, Virginia M. *et al*. (2014)^(82)^	Compassion fatigue, moral distress, and work engagement in surgical intensive care unit trauma nurses: A pilot study	Dimensions of Critical Care Nursing	113	United States
Watson, Jean; Smith, Marlaine C. (2002)^(40)^	Caring science and the science of unitary human beings: a trans-theoretical discourse for nursing knowledge development	Journal of Advanced Nursing	112	United States
Smith, Marlaine C. *et al.* (2002)^(83)^	Outcomes of therapeutic massage for hospitalized cancer patients	Journal of Nursing Scholarship	89	United States
Wiklund Gustin, Lena; Wagner, Lynne (2013)^(71)^	The butterfly effect of caring - clinical nursing teachers’ understanding of self-compassion as a source to compassionate care	Scandinavian Journal of Caring Sciences	89	United States
Perry, Beth (2009)^(84)^	Role modeling excellence in clinical nursing practice	Nurse Education in Practice	62	Canada
Baldursdottir, Gyda; Jonsdottir, Helga (2002)^(85)^	The importance of nurse caring behaviors as perceived by patients receiving care at an emergency department	Heart and Lung: Journal of Acute and Critical Care	59	Iceland
Wei, Holly; Watson, Jean (2019)^(43)^	Healthcare interprofessional team members’ perspectives on human caring: A directed content analysis study	International Journal of Nursing Sciences	36	United States
Cossette, Sylvie *et al.* (2006)^(51)^	A dimensional structure of nurse-patient interactions from a caring perspective: Refinement of the Caring Nurse-Patient Interaction Scale (CNPI-Short Scale)	Journal of Advanced Nursing	35	Canada

It is worth highlighting that the most influential journal, in relation to publications about Watson’s Theory of Human Caring, is the Journal of Advanced Nursing, which presents two of the eight most cited articles. Despite identifying Nursing Science Quarterly with the highest publication volume, presenting eight articles in its editions, the most cited journal was Dimensions of Critical Care Nursing, followed by the Journal of Advanced Nursing, with a volume of five publications in its editions, when compared to results in Charts 1 and 2. The references with the greatest impact were “Caring Science as Sacred Science”, with nine citations, “Postmodern Nursing and Beyond”, with 11 citations, and “Nursing: The Philosophy in the Science of Caring”, with 24 citations, all publications by Jean Watson. The countries with the greatest impact on publications were the United States, with 578 citations in 23 documents, followed by Canada, with 135 citations in six documents, Turkey, with 59 citations in ten documents, and Brazil, with 32 citations in 12 documents.

Authors with at least three articles indexed in the database and ten citations participated in the bibliographic coupling network, or scientific knowledge network, generating a network of ten authors, grouped into three clusters. Each researcher in a cluster tends to cite the same authors as the other participants in the same cluster.

The first cluster brought together four most productive researchers, Jean Watson and Marlaine Smith, from the University of Colorado Health Sciences Center, Denver, Colorado, with 112 citations, representing a strong connection to the other researchers in this network^(40)^. In the second cluster, consisting of three authors, Sylvie Cossete, Jose Cote, Jacinthe Pepin, Nicole Ricard and Louis-Xavier D’Aoust, Faculty of Nursing, University of Montreal, Quebec, Canada, in 2006 stand out, with 35 citations and eight sources of links with other researchers^(51)^. In the third cluster, formed by three authors, Anne Vitale had 21 citations and six connections in the network with the other researchers^(86)^. The studies discuss the implementation of systematized techniques for human care, highlighting the significant reduction in patients’ emotions and bad feelings integrated with technological care supports based on the Theory of Human Caring/Clinical Caritas Process. The authors highlight the Caritas Process as a nursing work model to strengthen the idea of holistic health care that is attentive to patients’ well-being as a whole. Therefore, they address implementation strategies to guarantee the quality of individual care and patient safety. It is possible to observe this reflection in the study entitled “Transformation of Clinical Nursing Practice Following a Caring-based Educational Intervention: A Qualitative Perspective”, by Bellier-Teichmann, Tanja *et al*.^(87)^.

It was possible to identify studies aimed at validating care instruments assessed by Watson’s theory of care. They also addressed the implementation of standardized systems in nursing services. These works discussed the importance of establishing an individual care relationship and developing clinical skills, using interaction scales and appropriate models for care. They emphasized that work engagement increases and stressors reduce individual satisfaction, resulting in environments supported by empathy, compassion and holism, as presented in the work entitled “Compassion fatigue, moral distress, and work engagement in surgical intensive care unit trauma nurses: A pilot study”, by Mason, Virginia M. *et al*.^(82)^.

Nursing care assessment based on Caritas-Veritas Clinical Process was another most debated topic among studies^(24, 31, 35, 37, 41, 42, 43, 55, 74, 77, 87, 88, 89)^. In the findings, the importance of developing processes for nursing education and training centered on the process of human care was identified, involving strategies for the advancement of nursing with the reintegration of essential activities that meet patients’ needs and guarantee quality of life.

When discussing nursing staff’s ethical behavior during patient care, the authors draw attention to the types of interpersonal relationships established with patients. They consider that clinical competence is important to emphasize the notion of human care, integrating environments and moments of care with patients and other nursing professionals^(68, 71)^.

## DISCUSSION

The main topics discussed were: nursing staff’s ethical behavior during patient care; theoretical-philosophical reflections on the theory of care and its advantages for nursing training; strategies to systematize nursing care through the implementation of standardized care techniques/protocols based on Caritas-Veritas Clinical Process; and validity of care instruments to record and measure Watson’s moment of nursing care.

In the last three years, there has been an increase in nursing studies focused on empathetic and holistic care, approaching the assumptions defended by Jean Watson^(14)^. The years 2020 and 2021 stand out due to the COVID-19 pandemic, a period that encouraged the production of studies regarding individualized patient care^(21, 22, 23, 24, 30, 31, 32, 33, 34, 35, 36, 37, 38, 43, 44, 45, 46, 48, 49, 56, 60, 80, 81, 82, 83, 84, 85, 86, 87, 88, 89, 90, 91, 92, 93)^.

This reflection is seen in consolidated studies carried out by the Observatory on Science, Technology and Innovation (OSTI), which verified a growth in scientific production of 32.2% in 2020. In the report issued, scientific productions between 2015 and 2020 were measured and compared. It was found that, in the face of the pandemic, the work presented by researchers contributed to the development of social references, diagnoses and treatments, expanding relevant knowledge for implementing strategies to combat the coronavirus^(94)^.

The high participation of publications carried out in the United States, Canada and Turkey corresponds to the percentage noted in this report regarding international collaboration^(94, 95, 96, 97, 98, 99)^. There is an interest in consolidating the concept of internationalization of science and establishing rapprochement between research centers and, with this, one can see the establishment of cooperation networks of references and study trend groups centered on terms such as “Caring”, “Nursing” and “Nursing Theory”.

The performance of the networks established in this analysis points to the tendency of knowledge production between countries regarding the Theory of Human Caring according to Watson and, therefore, they claim its importance as an approach in journal submissions. This statement is corroborated in the reflections made by Guerrero-Castañeda and Chávez-Urías^(22)^, who claim to be a theory used constantly in nursing processes, as its holistic perspective is centered on the construction of the values of interpersonal relationships, awakening a fundamental awareness for the professional practice of the area, which is care centered on patients and their needs.

The quality of nursing care can enhance the involvement of empathetic and holistic care, transcending technical and mechanized procedures. This is because the evolution of care recognizes the phenomenon of transpersonality between nurses and patients, and is understood as a manifestation of nursing professionalization maturation^(22)^. There is a tendency for nursing work to advance, and it is clear that the team’s ethical behavior during patient care is one of the topics most discussed by nursing professionals^(56, 90, 91, 92, 93)^. It is understood that care relationships during the nursing process must find a meaning capable of helping patients during their suffering. Therefore, it is important that nursing establishes relationships of patient acceptance in this moment of care^(98)^.

Ethical behavior during care awakens an intersubjective relationship between nurses and patients, since both are influenced by the stories, environment and phenomenon of care. By sharing the technical relationship of care, the moment of care transcends the environment and the physical moment to the spiritual state.

This type of behavior is exemplary and focused on care, being a reference for other nurses, as there is an effort to provide care to patients holistically and empathetically. Patients perceive nurses’ general care as something significant, based on changes in individual behaviors. In this sense, it is important to implement nurse education programs to provide patient care with quality nursing services^(55)^.

It is understood the importance of nursing professionals developing the ability to meet the needs related to the body-mind-spirit of patients, considering their particularities and, therefore, the importance of establishing ethical behavior in interpersonal relationships so that care is carried out intentionally and aligned with patients’ trust and beliefs^(81, 99)^.

The moment of care must be one in which nurses dedicate themselves to action and relationships with their patients and, therefore, constitute a moment with a structure of respect for those involved. Regarding theoretical-philosophical reflections on the theory of care and its advantages for nursing training, it is possible to observe the importance given to improving nursing knowledge and its development of care, in addition to the recurring suggestions to include theoretical assumptions in the curricula of undergraduate nursing courses. This approach is also seen in other literature, highlighting the tendency towards effective and interpersonal care^(81, 98, 99)^. This debate consists of facilitating, integrating and contributing to nursing work evolution and to patients’ healing and humanity. Effective care promotes the health and growth of patients and, therefore, the improvement of scientific nursing knowledge improves action at the time of care.

It is possible to find similar reflections in the literature that reinforce the need to rethink nursing education, highlighting the advantages of using educational technologies capable of stimulating professional identity empowerment by enhancing the apprehension of pertinent information and knowledge to develop specific skills^(99)^. This benefit can be used to strengthen the curriculum based on the Theory of Human Caring/Clinical Caritas Process as it allows sharing of instructions that improve the quality of the interpersonal relationship in nursing and the moment of care.

The International Council of Nurses (ICN) points out, in the description of nurses’ competencies, that they must have scientific knowledge and know the basic concepts of nursing science in their training. However, the meaning of the term care and its meaning for the profession is not unanimous in nursing education programs^(100)^.

The Theory of Human Caring/Clinical Caritas Process educational contributions highlight the realization of the nursing process through recovery and definition of concepts of involvement. It is a statement identified in the studies analyzed, and which corroborates other authors, since the theoretical basis values human aspects and contributes to better quality nursing care, considering the complexity of care and relationships with patients^(101)^. Reflections on patient-centered nursing training based on the Theory of Human Caring/Clinical Caritas Process support the evolution of scientific nursing knowledge, since knowledge production improves care practice^(98, 99)^.

There is a tendency to develop strategies to systematize nursing care through the implementation of standardized techniques or protocols for patient care centered on Caritas-Veritas Clinical Process without losing the importance of an individualized look at patients. The most recent debates about nursing staff’s behavior during individual patient care are focused on establishing spaces for human connection with patients, providing a moment of transpersonal care and incorporating Caritas-Veritas Clinical Process, such as intentional awareness, mindfulness, gratitude, purpose and spirituality during the nursing care process.

As observed in the study “COVID-19: An Organizational-theory-guided Holistic Self-caring and Resilience Project”^(45)^, by investing in infrastructure for nursing professionals’ holistic training, the ability to satisfy patient needs will improve care outcomes and establish better health quality indicators.

This reinforces the statements found in the literature that point to the importance of using instruments that involve the theory of care as a reference to disseminate efforts on the professional advancement of nursing^(99)^.

Finally, it is highlighted that Watson’s Theory of Human Caring contributes to nursing training and care, and Caritas-Veritas Clinical Process can be useful for the different possibilities of practice and teaching. Nursing must integrate a simple and transformative language during nursing practice, and transpersonal care can be a positive indicator for realizing empathetic and holistic care.

### Study limitations

The use of only one database for research is considered a parameter for the limits of results in this study. Furthermore, the term used is not linked in the DeCS and MeSH thesaurus database, highlighting the restriction of access to other publications on the topic studied.

### Contributions to nursing

The main contributions of this study aim to analyze the Theory of Human Caring/Clinical Caritas Process theoretical direction, given its evolution. The studies pointed to the use of theory in nursing care and teaching, providing more sensitive care. Although it is a theory created in 1979, it is still alive, in force and contributing to nursing care.

The methodological path adopted allowed us to map in depth the records and elements in which relationships of scientific knowledge are produced in the individual care field. Analysis helped identify trends and growth in production volume regarding knowledge of Watson’s Theory of Human Caring/Clinical Caritas Process, warning about the importance of advancing in the construction of science where knowledge gaps still exist. Furthermore, it provided records that link international reference networks and their relationships on the topic developed, meeting the most productive researchers on this topic.

## CONCLUSIONS

The study made it possible to identify a trend towards the production of works focused on the topic Theory of Human Caring/Clinical Caritas Process between 2020 and 2021, and clarified the collaboration network for research between institutions in different countries. These allowed us to trace the similarity between researchers, since scientific production is significant in the use of Jean Watson’s ideas to explain the evolution of her Care Theory. However, considering that only one database was used, there is little scientific production produced by the group of researchers from Latin America, requiring other studies in other databases, aiming at greater generation of empirical data about the implementation of the theory and consequently contributing to health care improvement for the population.

Hence, it was noticed that studies are focused on developing nursing staff’s ethical behavior through technical improvement, implementation and validity of instrument strategies capable of measuring and assessing the quality of holistic and empathetic care. Studies identified outlined a significant time frame and with an exponential increase during the COVID-19 pandemic, highlighting the importance of care theory when providing individual patient care and contributing to expansion of knowledge in the academic community.

## References

[1] 1 Daltro MR, Faria AA. Relato de experiência: uma narrativa científica na pós-modernidade. Estud Pesqui Psicol[Internet]. 2019 [cited 2023 Apr 31];19(1):223-37. Available from: http://pepsic.bvsalud.org/pdf/epp/v19n1/v19n1a13.pdf

[2] 2 Watson J. Elucidando a disciplina de Enfermagem como fundamental para o desenvolvimento da enfermagem profissional. Texto Contexto Enferm. 2017;26(4). 10.1590/0104-07072017002017editorial4

[3] 3 Alves HLC, Lima GS, Albuquerque GA. Uso das teorias de enfermagem nas teses brasileiras: estudo bibliométrico. Cogitare Enferm 2021;26:e71743. 10.5380/ce.v26i0.71743

[4] 4 Barros ALBL, Bispo GS. Teorias de enfermagem: base para o processo de enfermagem. In: Peres HHC, Maia FOMM, Gengo e Silva RC, Cruz DALM, coordenadoras do evento. I Encontro Internacional do Processo de Enfermagem (I ENIPE); 2017 Jun 22-23; São Paulo, Hospital Universitário da USP. São Paulo (Brasil): Galoá, Hospital Universitário da USP; 2017. p. 1-20. 10.17648/enipe-2017-85605

[5] 5 Rocha APS. Teoria de Imogene M. King. In: Souza DG, Brandão V P, Martins MN, Morais JAV, Jesus NO, organizadores. Teorias de enfermagem: relevância para a prática profissional na atualidade. Campo Grande: Inovar; 2021. p. 16.

[6] 6 Oliveira ALL. Teoria de Callista Roy. In: Souza DG, Brandão VP, Martins MN, Morais JAV, Jesus NO, organizadores. Teorias de enfermagem: relevância para a prática profissional na atualidade. Campo Grande: Inovar; 2021. p. 32-33.

[7] 7 Alves D P, Santos FA, Figueiredo HRPP, Tavares CMM. Empatia na assistência em enfermagem sob a luz de Watson. Rev Recien. 2021;11(36):629-635. 10.24276/rrecien2021.11.36.629-625

[8] 8 Sady AAB. Teoria de Martha Rogers. In: Souza DG, Brandão VP, Martins MN, Morais JAV, Jesus NO, organizadores. Teorias de enfermagem: relevância para a prática profissional na atualidade. Campo Grande: Inovar; 2021. p. 27.

[9] 9 Rocha APS. Teoria de Dorothea Orem. In: Souza DG, Brandão VP, Martins MN, Morais JAV, Jesus NO, organizadores. Teorias de enfermagem: relevância para a prática profissional na atualidade. Campo Grande: Inovar; 2021. p. 15.

[10] 10 Rodrigues AC. Teoria de Madeleine Leininger. In: Souza DG, Brandão VP, Martins MN, Morais JAV, Jesus NO, organizadores. Teorias de enfermagem: relevância para a prática profissional na atualidade. Campo Grande: Inovar; 2021. p. 21-22.

[11] 11 Watson J. Watson’s theory of human caring and subjective living experiences: carative factors/caritas processes as a disciplinary guide to the professional nursing practice. Texto Contexto Enferm. 2007;16(1):129-35. 10.1590/S0104-07072007000100016

[12] 12 Watson J. Nursing: the philosophy and science of caring. Boulder: University Press of Colorado; 2008.

[13] 13 Watson J. Enfermagem pós-moderna e futura: um novo paradigma da Enfermagem. Loures: Lusociência; 2002.

[14] 14 Clark CS. Watson’s Human Caring Theory: pertinent transpersonal and humanities concepts for educator. Humanities.; 2016;5(21). 10.3390/h5020021

[15] 15 Watson J. Caring science as sacred science. Boulder: Lotus Library; 2021.

[16] 16 Watson J. Human Caring and Human Science: a theory of nursing. Norwalk, CT: Appleton-Century-Crofts; 1985.

[17] 17 Watson J. Unitary caring science: the philosophy and praxis of nursing. Louisville, Colorado: University Press of Colorado, 2018.

[18] 18 Okubo Y. Bibliometric indicators and analysis of research systems: Methods and examples. French: OECD; 1997. 71 p. 10.1787/208277770603

[19] 19 Page MJ, Mckenzie JE, Bossuyt PM. A declaração PRISMA 2020: diretriz atualizada para relatar revisões sistemáticas. Rev Panam Salud Publica. 2022;46:e112. 10.26633/RPSP.2022.112 PMC9798848 36601438

[20] 20 Universiteit Leiden. VOSviewer: Visualizing scientific landscapes [Internet]. Holanda: Universiteit Leiden; 2023 [cited 2023 Mar 14]. Available from: https://www.vosviewer.com/

[21] 21 Christopher R, Tantillo L, Watson J. Academic caring pedagogy, presence and Communitas in nursing education during the COVID-19 pandemic. Nurs Outlook. 2020;68(6):822-9. 10.1016/j.outlook.2020.08.006 PMC7516667 32981671

[22] 22 Guerrero-Castañeda RF, Chávez-Urías RA. Momento de cuidado, un encuentro fenomenológico entre enfermera y persona cuidada: reflexión en Watson. Cult Cuid. 2020;24(58):7-18. 10.14198/cuid.2020.58.02

[23] 23 Othman F, Liu Y, Zhang X. Perinatal women’s satisfaction with nurses caring behaviours in teaching hospitals in China. Scand J Caring Sci. 2020;34(2):390-400. 10.1111/scs.12740 31334870

[24] 24 van der Westhuizen L, Naidoo K, Casmod Y, Mdlethse S. Sonographers’ experiences of being a caring professional within private practice in the province of Gauteng. Health SA Gesondheid. 2020;25:1409. 10.4102/hsag.v25i0.1409 PMC7736680 33354359

[25] 25 Molala W, Downing C. Experiences of newly qualified critical care nurses caring for post-cardiothoracic surgery pediatric patients in a private hospital in gauteng. IJANS. 2020;13:100213. 10.1016/j.ijans.2020.100213

[26] 26 Aghaei MH, Vanaki Z, Mohammadi E. Watson’s human caring theory-based palliative care: a discussion paper. Int J Cancer Manag. 2020;13(6):e103027. 10.5812/ijcm.103027

[27] 27 Linton M, Koonmen J. Self-care as an ethical obligation for nurses. Nurs Ethics. 2020;28;969733020940371. 10.1177/0969733020940371 32720570

[28] 28 Murali K P. End of Life Decision-Making: Watson’s Theory of Human Caring. Nurs Sci Q. 2020;33(1):73-78. 10.1177/0894318419881807 31795880

[29] 29 Perkins JB. Watson’s Ten Caritas Processes with the Lens of Unitary Human Caring Science. Nurs Sci Q. 2021;34(2):157-167. 10.1177/0894318420987176 33749435

[30] 30 Durgun Ozan Y, Duman M, Çiçek Ö, Baksi A. The effects of clinical education program based on Watson’s theory of human caring on coping and anxiety levels of nursing students: a randomized control trial. Perspect Psyc Care. 2020;56(3):621-28. 10.1111/ppc.12477 31970791

[31] 31 Gomes ET, Bezerra SMMS. Religiousness, Spiritual Well-Being and Transpersonal Caring in the Preoperative Period of Heart Surgery. Rev Cuid [Internet]. 2020 [cited 2023 Mar 25];11(2). Available from: https://revistas.udes.edu.co/cuidarte/article/view/1020

[32] 32 Griffin C, Oman KS, Ziniel SI. Increasing the capacity to provide compassionate care by expanding knowledge of caring science practices at a pediatric hospital. Arch Psychiatr Nurs. 2021;35(1):34-41. 10.1016/j.apnu.2020.10.019 33593513

[33] 33 Antonini M, Bellier-Teichmann T, O’Reilly L. Effects of an educational intervention to strengthen humanistic practice on haemodialysis nurses’ caring attitudes and behaviours and quality of working life: a cluster randomised controlled trial. BMC Nurs. 2021;20(1):255. 10.1186/s12912-021-00729-6 PMC8691052 34930206

[34] 34 Göral Türkcü S, Özkan S. The effects of reflexology on anxiety, depression and quality of life in patients with gynecological cancers with reference to Watson’s theory of human caring. Complement Ther Clin Pract. 2021;44:101428. 10.1016/j.ctcp.2021.101428 34157494

[35] 35 Gürcan M, Turan SA. Examining the expectations of healing care environment of hospitalized children with cancer based on Watson’s theory of human caring. J Adv Nurs. 2021;77(8):3472-82. 10.1111/jan.14934 34142737

[36] 36 Yang S, Guo W, Gong Y et al. Application of Vitamin A palmitate eye gel and nurse value of Watson’s theory of caring in children with dry eye after strabismus surgery: a randomized trial. Transl Pediatr. 2021;10(9):2335-46. 10.21037/tp-21-385 PMC8506051 34733674

[37] 37 Santos LB, Menezes TMO, Freitas RA, Sales MGS, Oliveira ALB, Nunes AMPB. Care for the spiritual dimension provided by caregivers in a nursing home. Rev Bras Enferm. 2021;75(1):e20200402. 10.1590/0034-7167-2020-0402 34586195

[38] 38 Evangelista CB, Lopes MEL, Costa SFG, Batista PSS, Duarte MCS, Morais GSN, et al. Nurses’ performance in palliative care: spiritual care in the light of Theory of Human Caring. Rev Bras Enferm. 2021;75(1):e20210029. 10.1590/0034-7167-2021-0029 34586201

[39] 39 Norred C. Minimizing preoperative anxiety with alternative caring-healing therapies. AORN J. 2000;72(5):838-40. 10.1016/s0001-2092(06)62015-2 11098363

[40] 40 Watson J, Smith MC. Caring science and the science of unitary human beings: a trans-theoretical discourse for nursing knowledge development. J Adv Nurs. 2002;37(5):452-61. 10.1046/j.1365-2648.2002.02112.x 11843984

[41] 41 Clarke PN, Watson J, Brewer BB. From theory to practice: caring science according to Watson and Brewer. Nurs Sci Q. 2009;22(4):339-45. 10.1177/0894318409344769 19858513

[42] 42 Özkan IA, Okumuş H, Buldukoǧlu K, Watson J. A case study based on Watson’s Theory of Human Caring: being an infertile woman in Turkey. Nurs Sci Q. 2013;26(4):352–9. 10.1177/0894318413500346 24085673

[43] 43 Wei H, Watson J. Healthcare interprofessional team members’ perspectives on human caring: A directed content analysis study. Int J Nurs Sci. 2019;6(1):17-23. 10.1016/j.ijnss.2018.12.001 PMC6608670 31406864

[44] 44 Penn AD, Phelps J, Rosa WE, Watson J. Psychedelic-Assisted Psychotherapy Practices and Human Caring Science: Toward a Care-Informed Model of Treatment. J Humanist Psychol. 2021;1-26. 10.1177/00221678211011013

[45] 45 Barnett P, Barnett M, Borgueta E. COVID-19: an Organizational-theory-guided Holistic Self-caring and Resilience Project. J Holist Nurs. 2021;39(4):325-35. 10.1177/08980101211007007 33861185

[46] 46 Gunawan J, Aungsuroch Y, Watson J, Marzilli C. Nursing Administration: Watson’s Theory of Human Caring. Nurs Sci Q. 2022;35(2):235-43. 10.1177/08943184211070582 35392719

[47] 47 Lukose A. Developing a practice model for Watson’s theory of caring. Nurs Sci Q. 2011;24(1):27-30. 10.1177/0894318410389073 21220572

[48] 48 Aktürk Ü, Erci B. The Effect of Watson’s Care Model on Anxiety, Depression, and Stress in Turkish Women. Nurs Sci Q. 2019;32(2):127-34. 10.1177/0894318419826257 30888300

[49] 49 Butcher HK. Unitary Caring Science: a hermeneutic-phenomenological research method. Nurs Sci Q. 2022;35(2):148-59. 10.1177/08943184211070593 35392720

[50] 50 Erci B, Sayan A, Tortumluoǧlu G. The effectiveness of Watson’s Caring Model on the quality of life and blood pressure of patients with hypertension. J Adv Nurs. 2003;41(2):130-9. 10.1046/j.1365-2648.2003.02515.x 12519271

[51] 51 Cossette S, Cote JK, Pepin J et al. A dimensional structure of nurse-patient interactions from a caring perspective: refinement of the Caring Nurse-Patient Interaction Scale (CNPI-Short Scale). J Adv Nurs. 2006;55(2):198-214. 10.1111/j.1365-2648.2006.03895.x 16866812

[52] 52 Cossette S, Pepin J, Côté JK. The multidimensionality of caring: a confirmatory factor analysis of the Caring Nurse-Patient Interaction Short Scale. J Adv Nurs. 2008;61(6):699-710. 10.1111/j.1365-2648.2007.04566.x 18302610

[53] 53 Bollinger E. Applied Concepts of Holistic Nursing. J Holist Nurs. 2001;19(2):212-4. 10.1177/089801010101900208 11847839

[54] 54 Wolf T P. Building a caring client relationship and creating a quilt: a parallel and metaphorical process. J Holist Nurs. 2003;21(1):81-7. 10.1177/0898010102250277 12666617

[55] 55 Lamke D, Catlin A, Mason-Chadd M. “Not Just a Theory”: the relationship between Jin Shin Jyutsu® Self-Care Training for Nurses and Stress, Physical Health, Emotional Health, and Caring Efficacy. J Holist Nurs. 2014;32(4):278-89. 10.1177/0898010114531906 24771664

[56] 56 Combs MA, Arnold T. Human Trafficking: empowering healthcare providers and community partners as advocates for victims. J Holist Nurs. 2022;40(3):295-301. 10.1177/08980101211045554 34569887

[57] 57 Urra ME, Jana AA, García MV. Some essential aspects of Jean Watson thought and her transpersonal caring theory. Cienc Enferm. 2011;17(3):11-22. 10.4067/s0717-95532011000300002

[58] 58 Salgado J, Valenzuela S, Sáez K. Nursing students and care receivers’ perceptions of caring behavior. Cienc Enferm. 2015;21(1):69-79. 10.4067/S0717-95532015000100007

[59] 59 Reis CC, Souza KRF, Alves DS. Women’s perception of their first labor experience: implications for nursing. Cienc Enferm. 2017;23(2). 10.4067/S0717-95532017000200045

[60] 60 Veras SMCB, Menezes TMO, Guerrero-Castañeda RF, Soares MV, Anton FR, Pereira GS. Nurse care for the hospitalized elderly’s spiritual dimension. Rev Bras Enferm. 2019;72(suppl 2):236-42. 10.1590/0034-7167-2018-0685 31826216

[61] 61 Norman V, Rossillo K, Skelton K. Creating healing environments through the theory of caring. AORN J. 2016;104(5):401-9. 10.1016/j.aorn.2016.09.006 27793250

[62] 62 Poblete-Troncoso MDC, Valenzuela-Suazo SV, Merino JM. Validation of two scales used to measure Transpersonal Human Caring, based on Jean Watson’s Theory. Aquichan. 2012;12(1). 10.5294/aqui.2012.12.1.1

[63] 63 Lagunes-Córdoba R, Hernández-Manzanares MA. A scale to assess the ethical conduct of nurses in patient care. Aquichan. 2012;12(3). 10.5294/aqui.2012.12.3.4

[64] 64 Delmas P, O’Reilly L, Cara C. Effects on nurses’ quality of working life and on patients’ quality of life of an educational intervention to strengthen humanistic practice among hemodialysis nurses in Switzerland: a protocol for a mixed-methods cluster randomized controlled trial. BMC Nurs. 2018;17:47. 10.1186/s12912-018-0320-0 PMC6249714 30479561

[65] 65 Suliman WA, Welmann E, Omer T, Thomas L. Applying Watson’s nursing theory to assess patient perceptions of bing cared for in a multicultural environment. J Nurs Res. 2009;17(4):293-7. 10.1097/JNR.0b013e3181c122a3 19955886

[66] 66 Boz I, Okumus H. The “Everything about the existence” experiences of Turkish women with infertility: solicited diaries in qualitative research. J Nurs Res. 2017;25(4):268-75. 10.1097/JNR.0000000000000166 28683014

[67] 67 Carson EM. Do performance appraisals of registered nurses reflect a relationship between hospital size and caring? Nurs Forum. 2004;39(1):5-13. 10.1111/j.0029-6473.2004.00005.x 15098320

[68] 68 Ranheim A, Kärner A, Berterö C. Caring theory and practice-entering a simultaneous concept analysis. Nurs Forum. 2012;47(2):78-90. 10.1111/j.1744-6198.2012.00263.x 22512765

[69] 69 Favero L, Pagliuca LMF, Lacerda MR. Transpersonal caring in nursing: an analysis grounded in a conceptual model. Rev Esc Enferm USP. 2013;47(2):500-5. 10.1590/s0080-62342013000200032 23743921

[70] 70 Santos MR, Bousso RS, Vendramim P, Baliza MF, Misko MD, Silva L. The practice of nurses caring for families of pediatric inpatients in light of Jean Watson. Rev Esc Enferm USP. 2014;48(especial):80-6. 10.1590/S0080-623420140000600012 25517839

[71] 71 Wiklund GL, Wagner L. The butterfly effect of caring - clinical nursing teachers’ understanding of self-compassion as a source to compassionate care. Scand J Caring Sci. 2013;27(1):175-83. 10.1111/j.1471-6712.2012.01033.x 22734628

[72] 72 Vianna ACA, Crossetti MGO. The movement between caring and caring yourself in ICU: an analysis through Watson’s Transpersonal Caring Theory. Rev Gaúcha Enferm [Internet]. 2004 [cited 2023 Mar 25];25(1):56-69. Available from: https://seer.ufrgs.br/index.php/rgenf/article/view/4494 15675566

[73] 73 Bergold LB, Alvim NAT. Musical visitation: therapeutic strategy based on theory of transpersonal caring. Online Braz J Nurs. 2008;7(1). 10.5935/1676-4285.20081469

[74] 74 Wu LM, Chin CC, Chen CH. Evaluation of a caring education program for Taiwanese nursing students: a quasi-experiment with before and after comparison. Nurse Educ Today. 2009;29(8):873-8. 10.1016/j.nedt.2009.05.006 19505747

[75] 75 Poirier P, Sossong A. Oncology patients’ and nurses’ perceptions of caring. Can Oncol Nurs J. 2010; 20(2):62-5. 10.5737/1181912x2026265 20572428

[76] 76 Ayala-Valenzuela R, Calvo-Gil MJ, Torres-Andrade MC, Koch-Ewertz T. Evidences for Watson phylosophy: preliminary version of the caring behaviors assessment in Chile. Rev Cuba Enferm. [Internet]. 2010 [cited 2023 Mar 25];26(1):42-51. Available from: http://scielo.sld.cu/pdf/enf/v26n1/enf08110.pdf

[77] 77 Santos MR, Silva L, Misko MD, Poles K, Bousso RS. Unveiling humanized care: nurses’ perceptions in pediatric oncology. Texto Contexto Enferm. 2013;33(3):646-53. 10.1590/S0104-07072013000300010

[78] 78 Santos I, França LR, Clos AC. Clinical process and comprehensive care in nursing people with cancer: Pilot study. Rev Enferm UERJ [Internet]. 2013 [cited 2023 Mar 25];21(1esp): 587-93. Available from: https://www.e-publicacoes.uerj.br/index.php/enfermagemuerj/article/view/10011/7805

[79] 79 Beaudoin MA, Ouellet Nicole. An exploration of factors influencing nursing practice with families experiencing perinatal loss. Rech Soins Infirm. 2018;(133):58-69. 10.3917/rsi.133.0058 30066508

[80] 80 Wood EB, Halverson A, Harrison G. Creating a Sensory-Friendly Pediatric Emergency Department. J Emerg Nurs. 2019;45(4):415-24. 10.1016/j.jen.2018.12.002 30679010

[81] 81 Costa JR, Arruda GO, Bareto MS, Poles K, Bousso RS. Cotidiano dos profissionais de enfermagem e Processo Clinical Caritas de Jean Watson: uma relação. Rev Enferm UERJ. 2019;27:e37744. 10.12957/reuerj.2019.37744

[82] 82 Mason VM, Leslie G, Clark K. Compassion fatigue, moral distress and work engagement in surgical intensive care unit trauma nurses: a pilot study. Dimens Crit Care Nurs. 2014;33(4):215-25. 10.1097/DCC.0000000000000056 24895952

[83] 83 Smith MC, Kemp J, Hemphill L, Vojir C P. Outcomes of therapeutic massage for hospitalized cancer patients. J Nurs Sch. 2002;34(3):257-62. 10.1111/j.1547-5069.2002.00257.x 12237988

[84] 84 Perry B. Role modeling excellence in clinical nursing practice. Nurse Educ Pract. 2009;9(1):36-44. 10.1016/j.nepr.2008.05.001 18590978

[85] 85 Baldursdottir G, Jonsdottir H. The importance of nurse caring behaviors as perceived by patients receiving care at an emergency department. Heart Lung. 2002;31(1):67-75. 10.1067/mhl.2002.119835 11805752

[86] 86 Vitale A. Nurses’ lived experience of Reiki for self-care. Holist Nurs Pract. 2009;23(3):129-41. 10.1097/01.HNP.0000351369.99166.75 19411991

[87] 87 Bellier-Teichmann T, Roulet-Schwab D, Antonini M. Transformation of Clinical Nursing Practice Following a Caring-based Educational Intervention: a qualitative perspective. SAGE Open Nurs. 2022;8:23779608221078100. 10.1177/23779608221078100 PMC8918767 35295618

[88] 88 Roulin MJ, Jonniaux S, Guisado H, Séchaud L. Perceptions of inpatients and nurses towards the importance of nurses’ caring behaviours in rehabilitation: a comparative study. Int J Nurs Pract. 2020;26(4):e12835. 10.1111/ijn.12835 32207212

[89] 89 Muslu L, Kolutek R, Fidan G. Experiences of COVID-19 survivors: a qualitative study based on Watson’s Theory of Human Caring. J Nurs Health Sci. 2022;24(3):774-84. 10.1111/nhs.12979 PMC9353283 35899855

[90] 90 Pajnkihar M, Stiglic G, Vrbnjak D. The concept of Watson’s carative factors in nursing and their (dis)harmony with patient satisfaction. Peer J. 2017;5:e2940. 10.7717/peerj.2940 PMC5299993 28194310

[91] 91 Sterchi S, Brooks S, Shilkaitis M, Ris L. Reconnecting nurses to their passion and enhancing the patient and family experience. J Nurs Adm. 2022;52(105):S48-S48. 10.1097/NNA.0000000000001203 31436740

[92] 92 Durgun Ozan Y, Çiçek Ö, Anuş Topdemir E. Experiences of nurses diagnosed with COVID-19 and recovered: a qualitative research. J Nurs Manag. 2022;10.1111/jonm.13825. 10.1111/jonm.13825 PMC9539309 36179722

[93] 93 Kurtgöz A, Koç Z. Effects of nursing care provided to the relatives of palliative care patients on caregivers’ spiritual well-being and hope: a randomized controlled trial. Omega (Westport). 2022;302228221124643. 10.1177/00302228221124643 36036673

[94] 94 Costa V. Produção brasileira de artigos cresce 32% em 2020 em relação a 2015 [Internet]. [São Paulo (BR)]: Sociedade Brasileira para o Progresso da Ciência; 2021 [cited 2023 Apr 19]. Available from: http://portal.sbpcnet.org.br/noticias/producao-brasileira-de-artigos-cresce-32-em-2020-em-relacao-a-2015/

[95] 95 Karlsson M, Pennbrant S. Ideas of caring in nursing practice. Nurs Philos. 2020;21(4):e12325. 10.1111/nup.12325 32876398

[96] 96 Petrou C. Guest Post: scientific output in the year of COVID. Scholarly Kitchen [Internet]. 2020 [cited 2023 Apr 19]. Available from: https://scholarlykitchen.sspnet.org/2020/11/19/guest-post-scientific-output-in-the-year-of-covid/

[97] 97 Centro de Gestão e Estudos Estratégicos. Panorama da ciência brasileira: 2015-2020. Boletim Anual OCTI [Internet]. 2021 [cited 2023 Apr 19];(1):196. Available from: https://www.cgee.org.br/documents/10195/11009696/CGEE_Pan_Cie_Bra_2015-20.pdf

[98] 98 Evangelista CB, Lopes MEL, Nóbrega MML. Análise da teoria de Jean Watson de acordo com o modelo de Chinn e Kramer. Rev Referência. 2020;5(4):e20045. 10.12707/RV20045

[99] 99 Santos IL, Nascimento LCN, Coelho MP, Freitas PSS, Moraes-Partelli AN. Educational material production and validity: educational instrument for home care for premature newborns. Rev Bras Enferm. 2023;76(1):e20210648. 10.1590/0034-7167-2021-0648pt PMC9885364 36722643

[100] 100 Sebrant L, Jong M. What’s the meaning of the concept of caring: a meta-synthesis. Scand J Caring Sci. 2021; 35:353-65. 10.1111/scs.12850 32271480

[101] 101 Dantas VPC, Evangelista CB, Vasconcelos MF. Publicações de teses e dissertações sobre a Teoria do Cuidado Humano: estudo bibliométrico. Rev Pesqui Cuid Fundam. 2021;13:822-8. 10.9789/2175-5361.rpcfo.v13.7590

