# Supplementary material for: Critical analysis of the scientific production on Jean Watson’s Theory of Human Care
Source: Rev Bras Enferm. 2024 Jun 14;77(2):e20230231. doi: 10.1590/0034-7167-2023-0231 (PMC11178309; doi:10.1590/0034-7167-2023-0231)
Supplement: Translation

# Translation: Análise crítica da produção científica sobre a Teoria do Cuidado Humano de Jean Watson

**DOI:** 10.1590/0034-7167-2023-0231pt

**Authors:** Shirley da Rocha Afonso, Maria Itayra Padilha, Vanessa Ribeiro Neves, Noe Ramírez Elizondo, Ricardo Quintão Vieira

**Affiliations:** I Universidade Federal de São Paulo. São Paulo, São Paulo, Brasil; II Universidade Federal de Santa Catarina. Florianópolis, Santa Catarina, Brasil; III Universidad de Costa Rica. San José, Costa Rica

**Keywords:** Cuidados de Enfermagem, Teoria de Enfermagem, Educação em Enfermagem, Modelos de Enfermagem, Métodos

## Abstract

**Objetivos:**

analisar a produção científica atual acerca da utilização dos preceitos teórico-metodológicos da Teoria do Cuidado Humano/*Processo Clinical Caritas* de Jean Watson.

**Método:**

estudo quantitativo, exploratório, descritivo, com recorte de 1979 a 2023 na base *Scopus*. Análise realizada com o *software VOSviewer*.

**Resultados:**

os 73 estudos incluídos estabelecem uma rede de colaboração entre 221 autores de 155 instituições em 18 países, que discutem o desenvolvimento do comportamento ético da equipe de enfermagem, por meio de aprimoramento técnico, implantação e validação de estratégias de instrumentos capazes de mensurar e avaliar a qualidade do cuidado holístico e empático. A Teoria do Cuidado Humano contribui para a formação e cuidado de enfermagem e *Process Clinical Caritas-Veritas* é útil para as diferentes possibilidades de prática e educação.

**Conclusões:**

importância em fortalecer com mais dados empíricos um modelo de trabalho de enfermagem centrado no cuidado humano individual, subsidiando a evolução do conhecimento científico da enfermagem.

## INTRODUÇÃO

A produção de conhecimento articula-se com a necessidade em desenvolver referenciais capazes de nortear o caminho do pensamento. Isso contempla um fazer distinto, capaz de consolidar as relações entre aqueles que produzem o conhecimento e aqueles que serão beneficiados por meio de ações de cuidado. A enfermagem é a profissão do cuidado, e esta é a base da ação desenvolvida em todos os ambientes de saúde em todo mundo^(1)^.

As bases teórico-filosóficas, valores e visões de mundo transcendem questões específicas, e buscam fornecer a compreensão universal acerca da realidade, além da reflexão ético-filosófica para todos os campos de estudo^(2)^. Neste sentido, entende-se que a enfermagem configura o seu saber com a multiplicidade da produção do conhecimento.

As teorias de enfermagem apontam para uma construção desse conhecimento múltiplo, fornecendo material teórico e prático na realização de ações diárias de cuidado. O conhecimento validado contribui para o avanço da profissão, uma vez que preenche a lacuna de debates e reflexões sobre o fazer da enfermagem. As teorias e conceitos consolidam as interrelações conceituais do próprio saber, estabelecendo e determinando uma relação lógica do pensamento crítico^(3,4)^.

Ao longo dos últimos 68 anos, várias estudiosas de enfermagem estabeleceram conceitos e teorias para explicar, instrumentalizar e sistematizar a compreensão de cuidado na prática cotidiana de enfermagem, como por exemplo, Virginia Henderson (Teoria das Necessidades Fundamentais, 1955), Faye Abdellah (Teoria Centrada nos Problemas, 1960), Ernestine Weidenbach (Teoria Prescritiva, 1964), Imogene M. King (Teoria dos Sistemas, 1964), Irmã Calista Roy (Teoria da Adaptação, 1964), Lydia Hall (Teoria do Cuidado Núcleo e Cura, 1966), Josephine Paterson e Loretta Zderad (Teoria da Enfermagem Humanística, 1966), Myra Estrin Levine (Teoria Holística, 1967), Wanda de Aguiar Horta (Teoria das Necessidade Humanas Básicas, 1970), Martha Rogers (Teoria do Ser Humano Unitário, 1971), Dorothea Orem (Teoria do Déficit do Autocuidado, 1971), Betty Neuman (Teoria de Sistema de Saúde, 1971), Madeleine Leininger (Teoria de Enfermagem Transcultural, 1978), Jean Watson (Teoria do Cuidado Humano, 1979), entre outras^(5,6,7,8,9,10,11)^.

Dentre essas, traça-se como foco deste estudo o pensamento e os pressupostos defendidos por Jean Watson. Reconhecer as ações disciplinadas pelas teorias como atividade intrínseca da profissão em enfermagem é admitir o estabelecimento da relação interpessoal na rotina de trabalho e, com isso, construir experiências sociais, emocionais e espirituais entre o enfermeiro e o paciente.

A filosofia de Watson é baseada em uma crença de que o cuidar é um ideal moral e não orientado para a tarefa, além de as pessoas cuidarem de si mesmas para cuidar dos outros. Watson vê a enfermagem como profissão e o cuidar como uma vocação baseada num acordo ético vinculado à humanidade^(12)^. Considera ainda, o cuidado como algo inovador, capaz de fortalecer a prática e liderança do enfermeiro no campo da saúde. Além disso, sua proposta de cuidado baseada na integração corpo-mente-alma é necessária como ferramenta apoiadora da experiência e compreensão do paciente e familiar sobre o processo de doença e cura^(13)^. O cuidado defendido por Watson exige uma conexão profunda entre o enfermeiro e o paciente durante todo os momentos de cuidado.

O cuidado é transpessoal e oportuniza a exploração dos sentimentos e emoções para além das ações técnico-procedimentais de enfermagem. As relações são interpessoais e promovem o vínculo entre corpo e mente, baseando-se na percepção sobre “*caring-healing*”^(14)^. Ou seja, as experiências adquiridas por meio das relações interpessoais entre o enfermeiro e paciente transcendem a percepção de ser, e adquirem uma consciência conectada com a pessoa cuidada e aquela que cuida. É uma ideia intersubjetiva que compartilha a história de vida de ambos os participantes no momento de cuidado^(13,14,15,16)^. A Teoria do Cuidado Humano fundamenta-se em um sistema de valores construídos na perspectiva holística e na visão unitária de mundo que se refere a uma experiência transpessoal do corpo, espírito e alma, criado no momento do cuidado, e pelas relações interpessoais entre o enfermeiro e o paciente. Nessa perspectiva, Jean Watson guia o cuidado transpessoal por meio do reconhecimento da consciência do “*healing*”. Esta consciência, denominada *Caritas*, é uma unidade que significa “estar presente no momento do cuidado”^(13,14,15,16)^.

Essa compreensão sobre o cuidado genuíno em um momento verdadeiro torna-se evidente na evolução da teoria a partir de 1980. Em 1985, o primeiro paradigma consistia em um cuidado baseado em fatores formados por um sistema de valores humanista-altruístas^(16)^. O segundo paradigma, em 2008, mostra uma abordagem ainda maior sobre o cuidado, quando passa a assumir uma consciência autêntica por meio de elementos que sustentam a relação transpessoal e, então, amplia-se para *Process Clinical Caritas* ^(12)^. Após 10 anos, Jean Watson apresenta seu terceiro paradigma, que envolve a transcendência do enfermeiro para uma consciência evoluída, aberta ao cósmico-divino e ao amor, acrescentando elementos-chave em um cuidado mais autêntico e expandindo para *Process Clinical Caritas-Veritas*. Nesse terceiro paradigma, Jean Watson afirma que o cuidado é um fenômeno único e reconhece que ele deve ocorrer de maneira unitária e, assim, propõe uma ampliação da compreensão sobre a evolução da Teoria do Cuidado Humano para a Ciência do Cuidado Unitário. Desta forma, a perspectiva de cuidado estabelece uma forma de perceber o mundo e relacionar-se de maneira a enfatizar o sentido transformador de cuidar e ser cuidado, ou seja, reconhecer a interação do relacionamento como uma experiência unitária do pensamento, corpo, alma e espírito durante o momento de cuidado^(17)^.

Com isso em mente, delineou-se a questão norteadora: qual é o panorama da produção científica internacional acerca da Teoria do Cuidado Humano de Jean Watson? É importante destacar que, ao compreender este panorama, será possível conservar e transmitir conhecimentos técnico-científico sobre o cuidado holístico e empático.

A justificativa deste estudo está na compreensão de que a finalidade em reunir experiências acerca das pesquisas sobre o cuidado humano auxiliará na proposição de novas perspectivas, dada a evolução de visão de mundo e das relações entre as pessoas que a teoria apresenta. Além disso, norteará novas pesquisas por meio da produção de conhecimentos científicos importantes para a evolução da prática de enfermagem baseada na teoria unitária e intencional, que enfatiza a conexão de experiências do cuidado entre enfermeiro e paciente.

## OBJETIVOS

Analisar a produção científica atual acerca da utilização dos preceitos teórico-metodológicos da Teoria do Cuidado Humano/Processo Clinical Caritas criada por Jean Watson.

## MÉTODOS

### Aspectos éticos

O estudo utilizou fontes documentais secundárias indexadas em base de dados e, por isso, não necessitou de aprovação em Comitê de Ética em Pesquisa.

### Tipo de estudo

Trata-se de um estudo com características quantitativas, exploratório e descritivo, com delineamento na Análise Bibliométrica^(18)^. Neste estudo foi aplicado um modelo híbrido por meio da sistematização de um conjunto de fases e etapas, buscando investigar o panorama, as aplicações e os resultados obtidos. Assim, o estudo foi dividido em duas fases, sendo 1. Busca na base de dados e 2. Análise Bibliométrica.

### Coleta e organização dos dados

A coleta de dados foi realizada na base de dados *Scopus*, sendo utilizada a Declaração PRISMA 2020 para apresentar a definição dos artigos científicos selecionados de modo sistemático^(19)^. A busca foi realizada no mês de março de 2023. A intenção de busca em uma única base de dados deu-se porque a base *Scopus* pode ser considerada uma base mais ampla, democrática, e conter um maior número de periódicos qualificados do que em outras bases.

Embora siga a orientação de um estudo bibliométrico, op-tou-se por realizar a busca da revisão sistemática a partir de uma pergunta de pesquisa, e adotou-se a estratégia PCC, utilizando o acrônimo para População (Enfermagem), Conceito (Preceitos teórico-metodológicos) e Contexto (Teoria do Cuidado Humano de Jean Watson), da qual foram identificados os conceitos e contextos dos seguintes termos nos resumos dos estudos a serem selecionados: *Fatores Caritativos, Modelo de Watson, Ciência do Cuidado da Watson, Ciência do Cuidado Humano de Watson, Ciência do Cuidado Unitário de Watson, Teoria do Cuidado de Watson ou Teoria de Watson ou Teoria do Cuidado Humano de Watson (Jean Watson) ou Teoria do Cuidado Transpessoal (Watson ou Jean Watson)*. Ao considerar o recorte histórico, optou-se pela busca de termos que expressassem a implementação da teoria desde 1979 até 2023, atendendo as várias concepções adotadas ao longo do tempo. Os critérios de inclusão foram: artigos científicos originais com abordagem sobre o cuidado do paciente baseado na Teoria do Cuidado Humano; as relações da teoria e a formação de enfermeiros; os processos de implantação da teoria no trabalho de enfermagem.

Como critérios de exclusão foram considerados, 1- as condições em que a teoria foi utilizada apenas para comparar com outra teoria de enfermagem; 2- abordagem conceitual sobre a teoria do cuidado, mas, sem o vínculo com a teoria defendida por Jean Watson; 3- trabalhos repetidos, as pesquisas de revisão (pesquisa secundária) e os trabalhos sem apresentação de resumos.

A primeira etapa Identificação consistiu em utilizar a estratégia de busca *TITLE-ABS-KEY (watson’s AND theory AND of AND caring) AND (LIMIT-TO (DOCTYPE, “ar”))*. O termo “Teoria do Cuidado de Watson” foi restrito aos campos título, resumo ou palavras-chave para localizar no relatório *Elsevier* 279 documentos indexadas na base de dados *Scopus* até a data de extração de informações, em 25 de março de 2023. A partir da implementação dos critérios de inclusão e exclusão, restaram 73 artigos para a análise da produção científica apresentados na Figura 1.

**Figura 1:** Fluxograma PRISMA 2020 para novas revisões sistemáticas que incluíram buscas apenas em bases de dados e registros

Fonte: PRISMA 2020.

### Análise dos dados

A diferença entre a quantidade de documentos localizados e artigos incluídos na análise evidencia a relevância da metodologia PRISMA para a produção de uma revisão sistemática e análises por meio de indicadores bibliométricos, pois permite a minimização do surgimento de vieses. A análise de dados foi realizada, inicialmente, pelo método da bibliometria, a qual identificou o cenário mais abrangente da literatura, e delineou a evolução e desenvolvimento do tema estudado. Neste caso, foi realizada por meio de indicadores quantitativos da amostra estatística descritiva para confecção de elementos gráficos. Os dados foram exportados em formato “*.*csv*” para análise bibliométrica em *software*, a fim de tratá-los estatisticamente por redes bibliométricas no programa VOSviewer versão 1.6.9^(18,20)^. Para organizar as informações qualitativas, os dados foram tabulados em arquivo *Microsoft Excel* 365 identificando os resumos, a autoria, ano de publicação, vínculo institucional, periódico, método utilizado e abordagem teórica. Essa organização constituiu num panorama da análise sistemática, permitindo localizar as lacunas e tendências das produções científicas.

## RESULTADOS

Inicialmente, foram identificados trabalhos publicados de 1979 a 2023, porém, depois da leitura dos resumos, foram selecionados aqueles que abordavam a Teoria do Cuidado Humano/Processo Clinical Caritas como escopo da investigação, o que restringiu a seleção aos anos 2000 a 2022. Não houve indexação de trabalhos na base de dados *Scopus* nos anos 2005 e 2007, contudo, não se pode afirmar o motivo da ausência de pesquisas sobre o tema nos periódicos durante este período. Identificou-se um aumento do número de publicações em periódicos indexados nos anos 2020 e 2021, evidenciando uma tendência na discussão sobre a Teoria. O ano de 2020, concentrou 11 publicações em diferentes periódicos^(21,22,23,24,25,26,27,28,29,30,31)^. Já o ano de 2021 registrou uma publicação em cada periódico, destaca-se a Revista Brasileira de Enfermagem com três artigos^(32,33,34,35,36,37,38)^.

Dos 73 artigos disponíveis com acesso completo, foi possível observar 221 autores associados, 155 instituições em 18 países, conforme Figura 2.

**Figura 2:** Distribuição geográfica de autores que desenvolveram estudos relacionados à Teoria do Cuidado de Watson

A figura 2 demonstra os países de origem dos autores que publicaram sobre o tema, sendo os Estados Unidos com 56 autores, destacando a Universidade do Colorado com oito autores; Brasil com 51 autores, com destaque para a Universidade Federal da Bahia, com nove autores; Turquia com 25 autores; Canadá com 17 autores; China com 15 autores; Chile; Suíça; com 12 autores cada país, África do Sul com seis autores; México com cinco autores; Arábia Saudita com quatro autores; Eslovênia, Irã e Taiwan com três autores cada país; Islândia, Tailândia e Finlândia com dois autores em cada país e Nova Zelândia com um autor. Foi possível identificar a evolução temporal das publicações sobre o termo pesquisado, destacando o número de ocorrências registradas em cada ano a partir de 2000, este primeiro sendo publicado por Caroline L. Norred^(38)^.

Dos 221 autores, cerca de 88% (195 autores) publicaram apenas um artigo, enquanto 11% (25 autores) publicaram dois a três artigos. Os Estados Unidos da América é um país com o maior volume de autores com publicações de trabalhos sobre o tema de estudo, totalizando 26 artigos indexados na *Scopus*, com destaque para Jean Watson (8 artigos)^(21,39,40,41,42,43,44,45,46)^.

Os 73 artigos que compuseram a amostra estão distribuídos em 47 periódicos. Ao observar a dispersão da produção científica sobre Teoria do Cuidado Humano de Watson, optou-se por organizar os periódicos em três grupos, considerando o total de publicações em cada. O primeiro grupo é composto por periódicos com o intervalo de três e oito publicações, e o segundo grupo com duas publicações cada. Já o terceiro grupo é constituído por periódicos que contemplam uma publicação cada. O Quadro 1 evidencia as fontes de publicação com maior volume de artigos na amostra investigada.

**Quadro 1:** Distribuição dos principais periódicos que divulgaram trabalhos sobre a Teoria do Cuidado de Watson, São Paulo, São Paulo, Brasil, 2023

Periódicos		Quantidade artigos	Localização
**Grupo 1 – 5 periódicos**		38	
*Nursing Science Quarterly* ^(28,29,41,42,46,47,48,49,50,51,52,53,54,55,56,57,58,59)^		8	Estados Unidos
*Journal of Advanced Nursing* ^(35,40,50,51,52)^		5	Estados Unidos
*Journal of Holistic Nursing* ^(42,53,54,55,56)^		5	Estados Unidos
*Ciencia y Enfermeria* ^(47,48,49,50,51,52,53,54,55,56,57,58,59)^		3	Chile
Revista Brasileira de Enfermagem^(37,38,60)^		3	Brasil
**Grupo 2 – 7 periódicos**		14	
*AORN Journal* ^(39,61)^		2	Estados Unidos
*Aquichan* ^(62,63)^		2	Chile
*BMC Nursing* ^(33,64)^		2	Suíça
*Journal of Nursing Research* ^(65,66)^		2	Arábia Saudita
*Nursing Fórum* ^(67,68)^		2	Estados Unidos
Revista da Escola de Enfermagem da USP^(69,68,69,70)^		2	Brasil
*Scandinavian Journal of Caring Sciences* ^(23,71)^		2	Estados Unidos
**Grupo 3 – 35 periódicos**		35	
**Total**	47 periódicos	73 artigos	

Em relação aos periódicos com maior volume de publicação destaca-se *Nursing Science Quarterly*, que representa 33,3% do grupo 1 de publicações na amostra de estudo, seguidos de *Journal of Advanced Nursing* e *Journal of Holistic Nursing* com 20,83% cada e os periódicos *Ciencia y Enfermeria* e Revista Brasileira de Enfermagem, que representam 12,5% cada dos periódicos com maior volume de trabalhos publicados. Os demais periódicos no grupo três somam 14,28% cada. O grupo 1 e 2 representam 52,05% do volume de publicação da amostra analisada^(72,73,74,75,76,77,78,79,80,81)^.

Foram elencadas, no Quadro 2, as publicações mais influentes sobre a temática, considerando o volume de citações recebidas por outros artigos publicados em periódicos indexados na base *Scopus*. Utilizou-se como critério de corte, o número mínimo de 35 citações realizadas entre a rede de citações internas dos artigos resultando em oito artigos que preencheram este critério, são eles:

**Quadro 2:** Volume de citações em artigos com maior impacto, São Paulo, São Paulo, Brasil, 2023

Autor(es) e Ano	Título	Periódico	Qtde de citações	Localização
Mason, Virginia M. *et al*. (2014)^(82)^	*Compassion fatigue, moral distress, and work engagement in surgical intensive care unit trauma nurses: A pilot study*	*Dimensions of Critical Care Nursing*	113	Estados Unidos
Watson, Jean; Smith, Marlaine C. (2002)^(40)^	*Caring science and the science of unitary human beings: a trans-theoretical discourse for nursing knowledge development*	*Journal of Advanced Nursing*	112	Estados Unidos
Smith, Marlaine C. *et al*. (2002)^(83)^	*Outcomes of therapeutic massage for hospitalized cancer patients*	*Journal of Nursing Scholarship*	89	Estados Unidos
Wiklund Gustin, Lena; Wagner, Lynne (2013)^(71)^	*The butterfly effect of caring - clinical nursing teachers’ understanding of self-compassion as a source to compassionate care*	*Scandinavian Journal of Caring Sciences*	89	Estados Unidos
Perry, Beth (2009)^(84)^	*Role modeling excellence in clinical nursing practice*	*Nurse Education in Practice*	62	Canadá
Baldursdottir, Gyda; Jonsdottir, Helga (2002)^(85)^	*The importance of nurse caring behaviors as perceived by patients receiving care at an emergency department*	*Heart and Lung: Journal of Acute and Critical Care*	59	Islândia
Wei, Holly; Watson, Jean (2019)^(43)^	*Healthcare interprofessional team members’ perspectives on human caring: A directed content analysis study*	*International Journal of Nursing Sciences*	36	Estados Unidos
Cossette, Sylvie *et al.* (2006)^(51)^	*A dimensional structure of nurse-patient interactions from a caring perspective: Refinement of the Caring Nurse-Patient Interaction Scale (CNPI-Short Scale)*	*Journal of Advanced Nursing*	35	Canadá

Vale destacar que o periódico mais influente, em relação às publicações acerca da Teoria do Cuidado de Watson, é *Journal of Advanced Nursing* que apresenta dois dos oito artigos mais citados. Apesar de identificar o periódico *Nursing Science Quartely* com maior volume de publicação, apresentando oito artigos em suas edições, o periódico mais citado foi o *Dimensions of Critical Care Nursing*, seguido do *Journal of Advanced Nursing* com um volume de cinco publicações em suas edições, quando comparados aos resultados dos Quadros 1 e 2. As referências com maior impacto foram os trabalhos *Caring Science as Sacred Science* com nove citações, *Postmodern Nursing and Beyond* e, com 11 citações, *Nursing: The Philosophy na Science of Caring* com 24 citações, todas publicações de Jean Watson. Os países com maior impacto nas publicações foram os Estados Unidos com 578 citações em 23 documentos, seguidos de Canadá com 135 citações em seis documentos, Turquia com 59 citações em 10 documentos e Brasil com 32 citações em 12 documentos.

Participaram da rede de acoplamento bibliográfico, ou rede de conhecimento científico, autores com pelo menos três artigos indexados na base de dados e 10 citações, gerando uma rede de 10 autores, agrupados em três clusters. Cada pesquisador integrante de um *cluster* tende a citar os mesmos autores que os demais participantes do mesmo *cluster*.

O primeiro *cluster* reuniu quatro pesquisadores mais produtivos, sendo Jean Watson e Marlaine Smith, da *University of Colorado Health Sciences Center* em Denver, Colorado, com 112 citações e representando uma forte ligação para os demais pesquisadores desta rede^(40)^. No segundo *cluster*, constituído por três autores, destacam-se Sylvie Cossete, Jose Cote, Jacinthe Pepin, Nicole Ricard e Louis-Xavier D’Aoust, da *FacultY of NursinG, UniversitY of Montreal*, Quebec, Canadá, em 2006, com 35 citações e oito fontes de ligações com os demais pesquisadores^(51)^. No terceiro *cluster*, formado por três autores, sendo Anne Vitale com 21 citações e seis ligações na rede com os demais pesquisadores^(86)^. Os estudos discutem sobre a implantação de técnicas sistematizadas para o cuidado humano, e destacam a redução significativa de emoções e sentimentos ruins dos pacientes integrados aos suportes tecnológicos de cuidados baseados na Teoria do Cuidado Humano/Processo *Clinical Caritas*. Os autores destacam o Processo *Caritas* como modelo de trabalho de enfermagem para fortalecer a ideia de cuidado em saúde holístico e atento ao bem-estar do paciente em sua totalidade. Por isso, abordam estratégias de implantação para garantir qualidade dos cuidados individuais e segurança do paciente. É possível observar essa reflexão no estudo *Transformation of Clinical Nursing Practice Following a Caring-based Educational Intervention: A Qualitative Perspective*, de Bellier-Teichmann, Tanja *et al.* ^(87)^.

Foi possível identificar estudos direcionados à validação de instrumentos de cuidados avaliados pela teoria do cuidado de Watson. Também abordaram a implantação de sistemas padronizados nos serviços de enfermagem. Estes trabalhos discutiram a importância em estabelecer uma relação de cuidado individual e desenvolvimento das habilidades clínicas, utilizando escalas de interação e modelos adequados para o cuidado. Enfatizaram que o engajamento para o trabalho aumenta e os fatores estressores diminui a satisfação individual, resultando em ambientes apoiados na empatia, compaixão e holismo, como apresentado no trabalho *Compassion fatigue, moral distress, and work engagement in surgical intensive care unit trauma nurses: A pilot study* de Mason, Virginia M. *et al.* ^(82)^.

A avaliação do cuidado de enfermagem baseada no *Process Clinical Caritas-Veritas* foi outro assunto mais debatido entre os estudos^(24,31,35,37,41,42,43,55,74,77,87,88,89)^. Nos achados, identificou-se a importância em desenvolver processos para a educação e formação de enfermeiros centrados no processo de cuidar humano, envolvendo estratégias para o avanço da enfermagem com a reintegração de atividades essenciais que satisfaçam as necessidades do paciente e garantam a qualidade vida.

Ao debaterem sobre o comportamento ético da equipe de enfermagem durante o cuidado do paciente, os autores chamam atenção sobre os tipos de relacionamentos interpessoais estabelecidos com os pacientes. Consideram que a competência clínica é importante para enfatizar a noção de cuidado humano, integrando os ambientes e momentos de cuidados com o paciente e outros profissionais de enfermagem^(68,71)^.

## DISCUSSÃO

Os principais temas debatidos foram o comportamento ético da equipe de enfermagem durante o cuidado do paciente; reflexões teórico-filosóficas a respeito da teoria do cuidado e suas vantagens para a formação de enfermeiros; estratégias para sistematizar a assistência de enfermagem por meio da implantação de técnicas/protocolos padronizados de atendimento, baseados no *Processes Clinical Caritas-Veritas*, e validação de instrumentos de cuidados para registrar e mensurar o momento de cuidado de enfermagem de Watson.

Nos últimos três anos, foram evidenciados um aumento de estudo de enfermagem centrados no cuidado empático e holístico, aproximando-se aos pressupostos defendidos por Jean Watson^(14)^. Destacam-se os anos 2020 e 2021, devido à pandemia COVID-19, um período que incentivou a produção de estudos a respeito do cuidado individualizado do paciente^(21,22,23,24,30,31,32,33,34,35,36,37,38,43,44,45,46,48,49,56,60,80,81,82,83,84,85,86,87,88,89,90,91,92,93)^.

Essa reflexão é percebida em estudos consolidados realizados pelo Observatório em Ciência, Tecnologia e Inovação (OCTI), que verificou um crescimento da produção científica em 32,2% no ano de 2020. No relatório emitido, foram mensuradas e comparadas as produções científicas entre 2015 e 2020, e constatou-se que, diante da pandemia, os trabalhos apresentados pelos pesquisadores contribuíram para o desenvolvimento de referências sociais, diag-nósticas e tratamentos, expandindo o conhecimento relevante para implantação de estratégias de enfrentamento do coronavírus^(94)^.

A alta participação das publicações realizadas nos Estados Unidos, Canadá e Turquia correspondem ao percentual percebido nesse relatório referente à colaboração internacional^(94,95,96,97,98,99)^. Há o interesse em consolidar o conceito de internacionalização da ciência e firmar a aproximação entre os centros de pesquisas e, com isso, percebe-se o estabelecimento de redes de cooperação de referências e grupos de tendências de estudos centrados em termos como *Caring, Nursing* e *Nursing Theory*.

O desempenho das redes estabelecidas nesta análise aponta a tendência de produção do conhecimento entre os países a respeito da Teoria do Cuidado Humano segundo Watson e, por isso, reivindicam sua importância de abordagem nas submissões de periódicos. Esta afirmação é corroborada nas reflexões realizadas por Guerrero-Castañeda e Chávez-Urías^(22)^, que afirmam ser uma teoria utilizada nos processos da enfermagem constantemente pois sua perspectiva holística é centrada na construção dos valores das relações interpessoais, despertando uma consciência fundamental para o exercício profissional da área, que é o cuidado centrado no paciente e em suas necessidades.

A qualidade do cuidado de enfermagem pode potencializar o envolvimento do cuidado empático e holístico, transcendendo os procedimentos técnicos e mecanizados. Isso porque, a evolução do cuidado reconhece o fenômeno da transpessoalidade entre o enfermeiro e o paciente, e é compreendida como uma manifestação de amadurecimento da profissionalização da enfermagem^(22)^. Há uma tendência para o avanço do trabalho da enfermagem, e percebe-se que o comportamento ético da equipe durante o cuidado com o paciente é um dos assuntos mais abordados pelos profissionais de enfermagem^(56,90,91,92,93)^. Entende-se que as relações de cuidado durante o processo de enfermagem devem encontrar um significado capaz de auxiliar o paciente durante seu sofrimento. Assim, é importante que a enfermagem estabeleça relações de aceitação do paciente nesse momento de cuidado^(98)^.

O comportamento ético durante o cuidado desperta para uma relação intersubjetiva entre o enfermeiro e o paciente, uma vez que ambos são influenciados pelas histórias, ambiente e fenômeno do cuidado. Ao partilhar a relação técnica do cuidado, o momento de cuidado transcende o ambiente e o momento físico para o estado espiritual.

Esse tipo de comportamento é exemplar e centrado no cuidado, sendo referência para outros enfermeiros, pois há um esforço para fornecer cuidados aos pacientes de forma holística e empática. Os pacientes percebem o cuidado geral dos enfermeiros como algo significativo, a partir da mudança de comportamentos individuais. Neste sentido, é importante a implantação de programas de educação de enfermeiros para atenderem ao paciente com qualidade do serviço de enfermagem^(55)^.

Entende-se a importância de o profissional de enfermagem desenvolver a capacidade em atender as necessidades relativas ao corpo-mente-espírito dos pacientes, considerando as suas particularidades e, portanto, a importância de estabelecer um comportamento ético na relação interpessoal, para que o cuidado seja realizado de forma intencional e alinhada com a confiança e as crenças dos pacientes^(81,99)^.

O momento do cuidado deve ser aquele em que o enfermeiro se dedica para ação e relação com seu paciente e, por isso, constitui em um momento com estrutura de respeito aos envolvidos. A respeito das reflexões teórico-filosóficas sobre a teoria do cuidado e suas vantagens para a formação do enfermeiro, é possível observar a importância dada em aprimorar o conhecimento da enfermagem e o seu desenvolvimento assistencial, além das recorrentes sugestões em incluir os pressupostos teóricos nos programas curriculares dos cursos de graduação em enfermagem. Tal abordagem é percebida também em outras literaturas, evidenciando a tendência para o cuidar eficaz e de maneira interpessoal^(81,98,99)^. Esse debate consiste em facilitar, integrar e contribuir para a evolução do trabalho de enfermagem e para a cura e humanidade do paciente. Um cuidado efetivo promove a saúde e o crescimento do paciente e, por isso, o aprimoramento do conhecimento científico da enfermagem melhora a ação no momento do cuidado.

É possível encontrar reflexões semelhantes na literatura que reforçam a necessidade de repensar a educação em enfermagem, destacando as vantagens da utilização de tecnologias educacionais capazes de estimular o empoderamento da identidade profissional por potencializar a apreensão de informações e conhecimentos pertinentes para o desenvolvimento de habilidades específicas^(99)^. Tal benefício pode ser utilizado em favor do fortalecimento do currículo baseado na Teoria do Cuidado Humano/Processo *Clinical Caritas*, por permitir o compartilhamento de instruções que melhoram a qualidade da relação interpessoal da enfermagem e do momento de cuidado.

O Conselho Internacional de Enfermeiros (ICN) aponta, na descrição das competências dos Enfermeiros, que estes devem ter conhecimento científico e saber os conceitos básicos da ciência da enfermagem em sua formação. Porém, não é unanimidade nos programas de educação em enfermagem o significado do termo cuidado e seu significado para a profissão^(100)^.

As contribuições educacionais da Teoria do Cuidado Humano/Processo *Clinical Caritas* destacam a realização do processo de enfermagem por meio de recuperação e definição de conceitos do envolvimento. É uma afirmação identificada nos estudos analisados, e que corroboram com outros autores, uma vez que o embasamento teórico valoriza os aspectos humanos e contribuem para um cuidado de enfermagem com melhor qualidade, considerando a complexidade do cuidado e das relações com paciente^(101)^. As reflexões sobre a formação de enfermagem centrada no paciente e embasada na Teoria do Cuidado Humano/Processo *Clinical Caritas* subsidiam a evolução do conhecimento científico da enfermagem, uma vez que a produção do conhecimento melhora a prática assistencial^(98,99)^.

Há uma tendência em desenvolver estratégias para sistematizar a assistência de enfermagem, por meio da implantação de técnicas ou protocolos padronizados para o cuidado ao paciente centrado no *Process Clinical Caritas-Veritas*, sem perder a importância do olhar individualizado sobre ele. Os debates mais recentes sobre o comportamento da equipe de enfermagem durante o cuidado individual do paciente estão concentrados em estabelecer espaços de conexão humana com o paciente, fornecendo um momento de cuidado transpessoal e incorporando o *Process Clinical Caritas-Veritas*, como consciência intencional, atenção plena, gratidão, propósito e espiritualidade durante o processo assistencial de enfermagem.

Conforme observado no estudo *COVID-19: An Organizational-theory-guided Holistic Self-caring and Resilience Project* ^(45)^, ao investir em infraestrutura para a formação holística dos profissionais de enfermagem, a capacidade de satisfazer o paciente melhorará os resultados de cuidado, e estabelecerá melhores indicadores de qualidade de saúde.

Isso reforça as afirmações encontradas na literatura que apontam para a importância do uso de instrumentos que envolvam a teoria do cuidado como referência para divulgar os esforços sobre o avanço profissional da enfermagem^(99)^.

Por fim, destaca-se que a Teoria do Cuidado de Watson contribui para a formação e assistência de enfermagem, e o *Process Clinical Caritas-Veritas* pode ser útil para as diferentes possibilidades da prática e ensino. É dever da enfermagem integrar uma linguagem simples e transformadora durante a prática de enfermagem e o cuidado transpessoal pode ser um indicador positivo para perceber o cuidado empático e holístico.

### Limitações do estudo

Considera-se parâmetro para os limites dos resultados neste estudo a utilização de apenas uma base de dados para investigação. Além disso, o termo utilizado não é vinculado no banco de *thesaurus* DECS e MESH, evidenciando a restrição de acesso às demais publicações sobre o tema estudado.

### Contribuição para a área da enfermagem

As principais contribuições deste estudo apontam para analisar o rumo teórico da Teoria do Cuidado Humano/Processo *Clinical Caritas*, dada a sua evolução. Os estudos apontaram o uso da teoria no cuidado e no ensino de enfermagem, sendo um cuidado mais sensível. Embora seja uma teoria criada em 1979, continua viva, em vigor e contribuitiva para o cuidado de enfermagem.

O caminho metodológico adotado permitiu mapear com profundidade os registros e elementos em que as relações do conhecimento científico são produzidas na área do cuidado individual. A análise auxiliou na identificação de tendência e crescimento do volume de produção acerca do conhecimento da Teoria do Cuidado Humano/Processo *Clinical Caritas* de Watson, alertando sobre a importância em avançar na construção da ciência em que ainda existem as lacunas do conhecimento. Além disso, ele forneceu registros que ligam as redes de referências internacionais e suas relações sobre o tema desenvolvido, conhecendo os pesquisadores mais produtivos neste tema.

## CONCLUSÕES

O estudo permitiu identificar uma tendência para a produção de trabalhos voltados para o tema Teoria do Cuidado Humano/Processo *Clinical Caritas* entre os anos 2020 e 2021, e clarificou a rede de colaboração para as pesquisas entre as instituições de diferentes países. Estas permitiram traçar a similaridade entre os pesquisadores, uma vez que a produção científica é significativa na utilização das ideias de Jean Watson para explicar a evolução da Teoria do Cuidado. Porém, considerando que foi utilizada apenas uma base de dados, percebe-se nesta uma escassa produção científica produzida pelo grupo de pesquisadores da América Latina, requerendo outros estudos em outras bases de dados, visando uma maior geração de dados empíricos acerca da implementação da teoria e consequentemente contribuir para a melhoria do cuidado a saúde a população.

Com isso, percebeu-se que os estudos são centrados no desenvolvimento do comportamento ético da equipe de enfermagem, por meio de aprimoramento técnico, implantação e validação de estratégias de instrumentos capazes de mensurar e avaliar a qualidade do cuidado holístico e empático. As publicações identificadas traçaram um recorte temporal significativo e com um aumento exponencial no período da pandemia de COVID-19, evidenciando a importância da teoria do cuidado no momento de cuidado individual do paciente e contribuindo para a ampliação do conhecimento na comunidade acadêmica.
